# Nobiletin and Xanthohumol Sensitize Colorectal Cancer Stem Cells to Standard Chemotherapy

**DOI:** 10.3390/cancers13163927

**Published:** 2021-08-04

**Authors:** Alice Turdo, Antonino Glaviano, Giacomo Pepe, Federica Calapà, Stefania Raimondo, Micol Eleonora Fiori, Daniela Carbone, Manuela Giovanna Basilicata, Veronica Di Sarno, Carmine Ostacolo, Barbara Parrino, Stella Cascioferro, Camilla Pecoraro, Simone Di Franco, Diana Bellavia, Miriam Gaggianesi, Veronica Veschi, Melania Lo Iacono, Gloria Ganduscio, Vincenzo Davide Pantina, Laura Rosa Mangiapane, Maria Rita Bongiorno, Riccardo Alessandro, Matilde Todaro, Ruggero De Maria, Patrizia Diana, Pietro Campiglia, Giorgio Stassi

**Affiliations:** 1 Department of Health Promotion, Mother and Child Care, Internal Medicine and Medical Specialties (PROMISE), University of Palermo, 90127 Palermo, Italy; alice.turdo@unipa.it (A.T.); laurarosa.mangiapane@unipa.it (L.R.M.); mariarita.bongiorno@unipa.it (M.R.B.); matilde.todaro@unipa.it (M.T.); 2 Department of Surgical, Oncological and Stomatological Sciences (DICHIRONS), University of Palermo, 90127 Palermo, Italy; antonino.glaviano@unipa.it (A.G.); simone.difranco@unipa.it (S.D.F.); miriam.gaggianesi@unipa.it (M.G.); veronica.veschi@unipa.it (V.V.); melania.loiacono@unipa.it (M.L.I.); gloria.ganduscio@unipa.it (G.G.); vincenzodavide.pantina@community.unipa.it (V.D.P.); 3 Department of Pharmacy, University of Salerno, 84084 Fisciano, SA, Italy; gipepe@unisa.it (G.P.); mbasilicata@unisa.it (M.G.B.); vdisarno@unisa.it (V.D.S.); 4 Fondazione Policlinico A. Gemelli, 00168 Rome, Italy; federica.calapa@guest.policlinicogemelli.it (F.C.); ruggero.demaria@unicatt.it (R.D.M.); 5 Department of BioMedicine, Neuroscience and Advanced Diagnostics (Bi.N.D), Biology and Genetics Section, University of Palermo, 90133 Palermo, Italy; stefania.raimondo@unipa.it (S.R.); riccardo.alessandro@unipa.it (R.A.); 6 Department of Oncology and Molecular Medicine (OMM), Istituto Superiore di Sanità (ISS), 00161 Rome, Italy; micol.fiori@iss.it; 7 Department of Biological, Chemical and Pharmaceutical Sciences and Technologies (STEBICEF), University of Palermo, Via Archirafi 32, 90123 Palermo, Italy; daniela.carbone@unipa.it (D.C.); barbara.parrino@unipa.it (B.P.); stellamaria.cascioferro@unipa.it (S.C.); camilla.pecoraro@unipa.it (C.P.); patrizia.diana@unipa.it (P.D.); 8 Department of Pharmacy, University of Naples Federico II, 80131 Napoli, Italy; carmine.ostacolo@unina.it; 9 Department of Molecular Medicine, Sapienza University, 00189 Rome, Italy; diana.bellavia@uniroma1.it; 10 Università Cattolica del Sacro Cuore, Istituto di Patologia Generale, 00168 Rome, Italy

**Keywords:** flavonoids, nobiletin, xanthohumol, anti-cancer therapy, cancer stem cells, colorectal cancer, natural biofunctional molecules

## Abstract

**Simple Summary:**

Colorectal cancer stem cells (CR-CSCs) play a pivotal role in the therapy resistance and relapse of CRC patients. Herein we demonstrate that new treatment approaches comprising polymethoxyflavones and prenylflavonoids extracted from *Citrus sinensis* and *Humulus lupulus*, respectively, hamper the viability of CR-CSCs as well as synergizing with 5-fluorouracil and oxaliplatin (FOX)-based chemotherapy. Extract fractions containing Nobiletin and Xanthohumol, in combination with chemotherapy, decreased stemness properties of CR-CSCs and restrained the outgrowth of chemoresistant metastatic CR-CSCs. These data pinpoint Nobiletin and Xanthohumol as efficacious anti-cancer compounds in metastatic settings.

**Abstract:**

Colorectal cancer (CRC) mortality is mainly caused by patient refractoriness to common anti-cancer therapies and consequent metastasis formation. Besides, the notorious toxic side effects of chemotherapy are a concurrent obstacle to be tackled. Thus, new treatment approaches are needed to effectively improve patient outcomes. Compelling evidence demonstrated that cancer stem cells (CSCs) are responsible for treatment failure and relapse. New natural treatment approaches showed capabilities to selectively target the CSC subpopulation by rendering them targetable by standard cytotoxic compounds. Herein we show the anti-cancer properties of the polymethoxyflavones and prenylflavonoids extracted from *Citrus sinensis* and *Humulus lupulus*, respectively. The natural biofunctional fractions, singularly and in combination, reduced the cell viability of CRC stem cells (CR-CSCs) and synergized with 5-fluorouracil and oxaliplatin (FOX) chemotherapy. These phenomena were accompanied by a reduced S and G2/M phase of the cell cycle and upregulation of cell death-related genes. Notably, both phytoextracts in combination with FOX thwarted stemness features in CR-CSCs as demonstrated by the impaired clonogenic potential and decreased Wnt pathway activation. Extracts lowered the expression of CD44v6 and affected the expansion of metastatic CR-CSCs in patients refractory to chemotherapy. Together, this study highlights the importance of polymethoxyflavones and prenylflavonoids as natural remedies to aid oncological therapies.

## 1. Introduction

Colorectal cancer (CRC) is the third most common cancer worldwide and the second most deadly cancer with a mortality rate of 600.000 deaths every year [1]. Most importantly, the 5-year survival rate ranges from 90 to 10% for stage I and stage IV patients, respectively [2]. The current clinical practice of cancer treatment mainly relies on surgery, chemotherapy, and radiotherapy. These approaches may be helpful to patients to some extent; however, more in-depth research is urgently needed to establish an unmet resolving therapy. The threatening fact of CRC is that despite some initial responses to currently available treatments, most patients with advanced stages could succumb to the disease due to therapy resistance and metastasis formation [3]. Therefore, determining the molecular mechanism of CRC resistance is crucial for designing new effective strategies.

Notably, a subpopulation of CRC cells, commonly identified by the expression of cell surface markers CD133 and CD44v6, are endowed with stemness properties, and thus defined as CRC stem cells (CR-CSCs) [4,5]. Remarkably, these cells play a critical role in the metastasis and relapse of CRC since they feature intrinsic properties of tumorigenesis, invasion, metastasis formation, and therapy resistance [5,6]. The underlying mechanism of CR-CSC resistance to treatment includes the activation of stemness signaling pathways, such as Wnt/β-catenin [7], Hedgehog [8], Notch [9], Hippo/Yap [10], and PI3K/AKT [5], as well as the high activity of detoxifying enzymes, and the increase of drug efflux pump levels [11] and anti-apoptotic factors [12].

During the last century, we have witnessed the use of chemotherapy as a synthetic drug-based treatment of cancer, which has improved the overall quality and extension of patients’ lives [13]. The addition of the third-generation platinum derivative oxaliplatin to a regimen of 5-FU and leucovorin (folinic acid) (FOLFOX), has become the mainstay of therapy in postoperative patients and metastatic CRC [14]. The FOLFOX regimen has been shown in multiple trials to improve progression-free survival (PFS) and overall survival (OS), compared with 5-FU and leucovorin alone, with more than 50% of response rates [15]. Of note, the non-chemotherapeutic drug leucovorin increases the anti-cancer effects of fluorouracil, while reducing the side effects caused by fluorouracil plus oxaliplatin (FOX) [16].

Notwithstanding the fact that chemotherapy is one of the major pharmacological therapy for cancer [17], toxicity to normal cells have hampered its current efficacy [18]. Side effects firstly reduce patient’s quality of life and also compromise therapy efficacy due to drug discontinuation and dose reduction [19]. Inevitably, the most common adverse effects reported for FOLFOX are tiredness and fatigue, organ toxicity, myelosuppression, and elevated liver transaminase levels [20]. Hence, due to the aforementioned reasons, reducing the undesired toxicity by selective protection of healthy cells without compromising the killing of transformed cells represents the only promising strategy to enhance CRC treatment.

Interestingly, regular consumption of fruits and vegetables is known to be protective against the risk of numerous cancers [21]. In line with this, during the last decades, there has been growing evidence of an inverse association between citrus fruit intake [22], as well as hop intake [23], and risk of cancer. Consequently, there has been an increasing interest in exploiting the potential role of citrus and hop in preventing or treating cancer [24], along with their possible use in combination with oncological therapies [25].

The tumor preventive effect of orange and hop is mainly exerted by biologically active polyphenols, such as flavonoids, which display antioxidant activity, control cell cycle progression, and modulate the activation of oncogenic pathways [26]. Among flavonoid subgroups, two well-known anticancer molecules derived from orange and hop are respectively Nobiletin [27] and Xanthohumol [28]. In CRC cell lines, the polymethoxyflavone Nobiletin inhibits proliferation, induces cellular apoptosis, limits angiogenesis, sensitizes cells to chemotherapy, and prevents tumor formation [29]. Similarly, the prenylflavonoid Xanthohumol inhibits cell proliferation, induces DNA damages and apoptosis, and sensitizes CRC cell lines to chemotherapy [30]. However, the use of polyphenols in clinical practice has been limited by a lack of knowledge regarding their long-lasting anti-cancer properties and the therapeutic doses avoiding cytotoxicity against normal cells. Moreover, purification of Nobiletin and Xanthohumol from the phytocomplexes or its chemical synthesis remains quite expensive, and multi-kilograms-scale production is far to come [31,32], thus new strategies should be implemented to achieve potential therapeutic use of these molecules. Bioactivity-based fractionation of natural extracts represents a time- and cost-saving approach [33,34] and it is particularly relevant from the point of view of a sustainable economy, allowing waste recovery [35,36].

Herein we show that selected fractions from Citrus sinensis and Humulus lupulus extracts, containing Nobiletin (NCF) and Xanthohumol (XCF) as the main components, respectively, decrease the cell viability of primary cells isolated from CRC patients and CRC cell lines, causing reduced and negligible cytotoxicity toward healthy cells at therapeutic concentrations. In addition, these fractionated extracts, either singularly or in combination, may synergize with FOX-based chemotherapy to increase apoptosis, impair the sphere-forming capability, reduce the S phase and the G2/M phase of the cell cycle, and decrease the activation of the Wnt pathway as well as the expression of the metastatic CR-CSC marker CD44v6. Notably, both fractions exerted a cytotoxic effect against CR-CSCs isolated from liver metastasis of chemoresistant patients, sensitizing them toward standard chemotherapy.

This study implies that the strategy of rational fractionation of natural extracts may represent a promising forefront remedy to improve future CRC chemotherapy, by both enhancing drug efficacy to reduce CSC survival as well as decreasing drug cytotoxicity. 

## 2. Materials and Methods

### 2.1. Sample Preparation

Hand-squeezed juice of Citrus sinensis var. Tarocco was centrifuged at 15,000× *g* for 15 min at 25 °C to remove fibers, then lyophilized for 24 h at −52 °C (LyoQuest-55, Telstar Technologies, Terrassa, Spain). The powder thus obtained was extracted with MeOH (Sigma-Aldrich, St. Louis, MO, USA) and the procedure was repeated three times for the complete recovery of a polyphenolic fraction [37].

Hop pellets were converted to powder with a mortar and treated with hexane for 10 min and then extracted with MeOH for 10 min (×3) [38].

The methanolic extracts were combined, evaporated to dryness under vacuum at 40 °C in a rotary evaporator, dissolved in MeOH:water 50:50 (*v*/*v*) to a concentration of 1 mg mL^−1^, filtered on a 0.45 µm nylon membrane (Merck Millipore, Milan, Italy), and finally analyzed by reverse phase (RP)-ultra-high performance liquid chromatography (UHPLC) coupled to diode array detection (DAD) and mass/mass spectrometry (MS/MS).

### 2.2. LCMS–IT-TOF Conditions

UHPLC-ESI-IT-TOF analyses were performed on a Shimadzu Nexera UHPLC system coupled online to an LCMS–IT-TOF mass spectrometer through an ESI source (Shimadzu, Kyoto, Japan). LC-MS data elaboration was performed by the LCMSsolution^®^ software (Version 3.50.346, Shimadzu). LC-MS analysis of polyphenolic compounds was carried out on a Kinetex^TM^ EVO C18 150 × 2.1 mm × 2.6 μm (100 Å) column thermostated at 40 °C (Phenomenex, Bologna, Italy), monitoring the chromatograms at 330 and 370 nm. Mobile phases consisted of 0.1% (*v*/*v*) CH_3_COOH/H_2_O (A) and 0.1% (*v*/*v*) CH_3_COOH/ACN (B). Analysis was performed in gradient elution as follows: 0–25 min, 15–90%B; 25–27.0 min, isocratic to 90%B; then five minutes for column re-equilibration. MS detection of polymethoxyflavones (*Citrus sinensis*) and prenylflavonoids (*Humulus lupulus*) was operated in positive ionization and a negative mode, respectively, with the following parameters: Detector voltage, 1.55 kV; CDL (curve desolvation line) temperature, 250 °C; block heater temperature, 250 °C; nebulizing gas flow (N_2_), 1.5 L min^−1^; drying gas pressure, 110 kPa. Full scan MS data were acquired in the range of 150–2000 *m*/*z* and MS/MS experiments were conducted in a data-dependent acquisition, while precursor ions were acquired in the range 150–1000 *m*/*z*.

Molecular formulas of identified compounds were calculated by the Formula Predictor software (Version 1.12, Shimadzu). The following online databases were also consulted: ChemSpider (http://www.chemspider.com, accessed on 12 May 2021), SciFinder Scholar (https://scifinder.cas.org, accessed on 12 May 2021) and Phenol-Explorer (www.phenol-explorer.eu, accessed on 12 May 2021).

### 2.3. Semiprep-RPHPLC-UV/Vis

The purification of polymethoxyflavones and prenylflavonoids was carried out by semi-preparative reversed-phase liquid chromatography employing a Shimadzu Semiprep-HPLC system consisting of two LC20AP pumps, a SIL20AP autosampler, a fraction collector FRC10A, a UV detector SPD20AV equipped with a preparative cell, and a system controller CBM 20A.

The separation was carried out on a Kinetex^TM^ C18 150 × 21.2 mm × 5 μm (100 Å), employing water (A) and acetonitrile (B) as mobile phases, both acidified by 0.1% (*v*/*v*) CH_3_COOH setting the flow rate at 20 mL min^−1^. The analysis was performed in gradient elution as follows:

*Citrus sinensis* gradient: 0–30 min, 10–70%B; 30–35 min, 70–10%B; 35–40 min, isocratic to 10%B.

*Humulus lupulus* gradient: 0–15 min, 5–30%B; 15–20 min, 30–70%B; 20–22 min, 70–100%B; 22–27 min, isocratic to 100%B; then five minutes for column re-equilibration.

### 2.4. Cell Culture

The purification and culture of CSphCs, from 6 primary tumor specimens and 6 liver metastasis of patients diagnosed with CRC, were performed as described in [39], in accordance with the ethical standards of Human Experimentation (authorization CE9/2015, Policlinico “Paolo Giaccone”, Palermo and authorization AIRC IG 2015, 17621, 2016, Fondazione Policlinico A Gemelli IRCCS, Rome, Italy). The authentication of CR-CSphCs is routinely performed by the short tandem repeat (STR) DNA profiling kit (GlobalFiler™ STR kit, Applied Biosystem, Thermo Fisher Scientific, Waltham, MA, USA) followed by sequencing analysis on ABIPRISM 3130 (Applied Biosystem, Thermo Fisher Scientific Waltham, MA, USA). Mycoplasma infection is constantly monitored with the MycoAlert TM Plus Mycoplasma Detection Kit (Lonza, Houston, TX, USA). DNA profiles of patient tumor tissues were matched with the corresponding CR-CSphCs.

HCT116 and RKO CRC cell lines were purchased by ATCC (Manassas, VA, USA) and cultured in DMEM (Sigma-Aldrich, St. Louis, MO, USA) supplemented with 10% FBS (Corning, Corning, NY, USA). HUVEC and HS-5 cell lines were purchased by ATCC (Manassas, VA, USA) and cultured in the Vascular Cell Basal Medium supplemented with the Vascular Endothelial Cell Growth Kit-VEGF (ATCC, Manassas, VA, USA) and in DMEM (ATCC, Manassas, VA, USA) supplemented with 10% FBS, respectively.

CRC cells were treated with 5-fluorouracil (Selleckchem, Houston, TX, USA) plus oxaliplatin (Sigma-Aldrich, St. Louis, MO, USA). Oxaliplatin was administered 3 h before 5-fluorouracil.

### 2.5. Cell Viability

Cell viability was determined by adding the CellTiter 96 AQueous One Solution Reagent (Promega, Madison, WI, USA) to untreated and treated CR-CSCs and CRC cell lines. The solution was incubated for 2 h at 37 °C and the 490 nm absorbance was assessed by using the Programmable MPT plate reader (GVD).

Cell viability was assessed with the MTT (3-(4,5-dimethylthiazol-2-yl)-2,5-diphenyltetrazolium bromide) in the two normal cell lines, HUVEC and HS5. Twenty-four hours after seeding, the cells were treated with 5–10 and 25 µg/mL of orange fractionated extract, hop fractionated extract, and mix extract. After 24 or 48 h treatment, MTT was added to each well and the plate was incubated for 3 h at 37 °C. After the addition of isopropanol, the plate was read at 540 nm.

### 2.6. Drug Combination Study

Drug combination studies have been assessed by using the Chou–Talalay method, which is based on the median effect and the combination index (CI) equations in order to determine the quantization of drug interactions. The CI), computed in CompuSyn using the Chou–Talalay method, calculated on cell proliferation following the treatment with different FOX and Nobiletin dose pairs. CI < 1 represented synergism (slight, moderate, strong, very strong); otherwise, it indicated additivity (CI = 1) or antagonism (CI > 1) between two drugs [40].

### 2.7. Transfection of Cells, Lentiviral Particle Production, and Cell Transduction

In order to produce lentiviral particle HEK-293T, packaging cells were transfected with TOP-GFP (Addgene, Watertown, MA, USA), psPAX2 (Addgene), and pMD2.G (Addgene) using XtremeGENE HP DNA Transfection Reagent (Roche, Basel, Switzerland). Lentiviral particles were subsequently concentrated by using the Lenti-X Concentrator reagent (Clontech, Takara Bio, San Jose, CA, USA). CR-CSphCs were transduced with the lentiviral particle and 8 μg/mL of polybrene (Sigma-Aldrich, St. Louis, MO, USA).

### 2.8. Clonogenic and Sphere Forming Assay

CR-CSphCs were pretreated for 48 h with NCF, XCF, Mix, and FOX chemotherapy and subsequently plated as single cells per well in a 96-well plate. Wells containing 1, 2, 3, 4, or 5 cells were included in the analysis. CR-CSphCs clonogenic potential was calculated with the Extreme Limiting Dilution Analysis (ELDA) ‘limdil’ function (http://bioinf.wehi.edu.au/software/elda, accessed on 2 April 2021).

In order to assess the sphere-forming capability of CR-CSCs, single cells, pretreated for 48 h with NCF, XCF, Mix, and FOX chemotherapy, were plated in ultra-low 6-well plates at 5.000 and 10.000 cells/mL density [41]. The sphere counts were performed after 48 h by using ImageJ software. The dense and tightly compacted structures were considered spheres. Sphere-forming efficiency was calculated with the formula (number of spheres/number of seeded cells) × 100.

### 2.9. RNA Isolation and Gene Expression Analysis

The purification of RNA was carried out using TRIZOL (Thermo Fisher Scientific, Waltham, MA, USA) protocol. For gene expression analysis, the total RNA (1 µg) was retrotranscribed and subjected to quantitative real-time PCR (qRT-PCR) with a custom PrimePCR panel (Bio-Rad, Hercules, CA, USA) for 88 genes involved in cell death, stemness, and the epithelial-to-mesenchymal transition according to the manufacturer’s instructions. Single gene assays were also performed using an SYBR green PCR mastermix (Qiagen, Hilden, Germany) and the following primers: *DKK1* (forward: 5′- GGT ATT CCA GAA GAA CCA CCT TG -3′; reverse: 5′- CTT GGA CCA GAA GTG TCT AGC AC -3′); *WNT5B* (forward: 5′- CAA GGA ATG CCA GCA CCA GTT C -3′; reverse: 5′- CGG CTG ATG GCG TTG ACC ACG -3′); *WNT3A* (forward: 5′- ATG AAC CGC CAC AAC AAC GAG G -3′; reverse: 5′- GTC CTT GAG GAA GTC ACC GAT G -3′); *WNT7B* (forward 5′- AGA AGA CCG TCT TCG GGC AAG A -3′; reverse 5′- AGT TGC TCA GGT TCC CTT GGC T -3′). The mRNA level was normalized to *GAPDH* (forward: GCT TCG CTC TCT GTC CCT CCT GT; reverse: TAC GAC CAA ATC CGT TGA CTC CG) housekeeping gene and calculated using the CT comparative method (ΔΔCt method).

### 2.10. Flow Cytometry

CR-CSCs were washed in PBS twice, and stained for 1 h at 4 °C with conjugated antibodies CD44v6-APC (2F10, mouse IgG1, R&D systems, Minneapolis, MN, USA) or isotype-matched control (IC002A, mouseIgG1, R&D systems, Minneapolis, MN, USA). Dead cells were excluded based on the uptake of 7-AAD (BD Biosciences, Franklin Lakes, NJ, USA).

For cell cycle analysis, untreated and treated CR-CSC were washed with PBS and centrifuged at 1300 rpm for 5 min. After removing the supernatant, the cell pellet was resuspended in 1 mL of Nicoletti Buffer (0.1% of Sodium citrate 0.01% of Tritox-100, 50 μg/mL of Propidium Iodide, 10 μg/mL of Rnase solution) and incubated in the dark at 4 °C for 16 h.

Apoptotic cells were detected by using the CaspGlow Fluorescein Active Caspase 3 Staining kit (Biovision, Milpitas, CA, USA) and Brilliant Violet 421 Annexin V apoptosis staining kit (Biolegend, San Diego, CA, USA) according to the manufacturer’s protocol. CR-CSCs were then analyzed using the FACSLyric flow cytometer (BD Biosciences, Franklin Lakes, NY, USA).

## 3. Results

### 3.1. Nobiletin and Xanthohumol Hamper CR-CSphCs Viability While Sparing Healthy Cells

Our group and others have previously demonstrated that CR-CSCs possess the capability to withstand chemotherapy and guide disease recurrence [39]. We sought to investigate to what extent the standard anti-cancer approach based on chemotherapy counteracted the viability of our CRC spheroid cells (CR-CSphCs) collection, which are heterogeneous cell populations composed of CSC, transit-amplifying, and differentiated cells (Table S1) [42].

**Figure 1:**
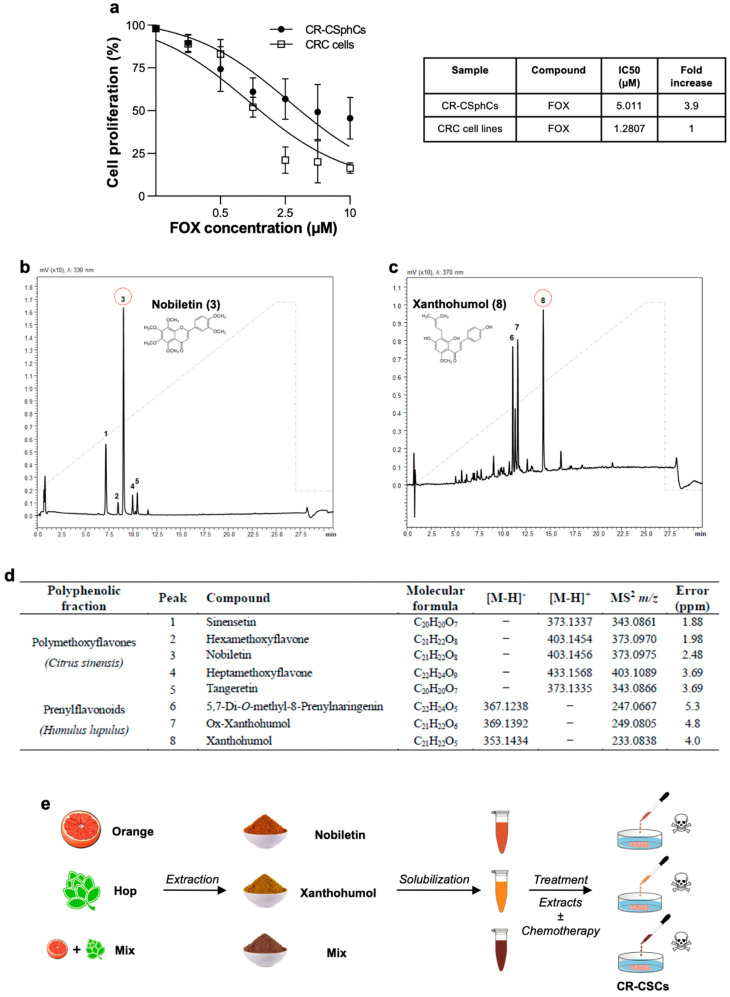
CR-CSphCs are resistant to chemotherapy. (**a**) Cell proliferation percentage of CR-CSphCs (#3, #8, and #59) and CRC cell lines (HCT116 and RKO) treated with 0.1 µM, 0.5 µM, 1 µM, 2.5 µM, 5 µM, and 10 µM of FOX chemotherapy for 48 h. Data are represented as mean ± SD of three independent experiments. IC50 values are indicated in the right panel; (**b**) chromatographic profiles of polymethoxyflavones and chemical structure of Nobiletin, isolated from *Citrus sinensis;* (**c**) chromatographic profiles of prenylflavonoids and chemical structure of Xanthohumol, isolated from *Humulus lupulus;* (**d**) qualitative profile of isolated polyphenolic fractions; (**e**) NCF, XCF, and Mix (50% NCF plus 50% XCF) were firstly extracted through lyophilization and then solubilized in methanol. CR-CSphCs and CRC cell lines were exposed to extracts, either singularly or in combination with FOX chemotherapy.

Following exposure to increasing concentrations of 5-fluorouracil and oxaliplatin (FOX)-based therapy, by mimicking the clinically used schedule and doses for FOX [15,43], CR-CSphCs showed higher resistance to this therapeutic regimen as compared to established CRC cell lines (Figure 1A). Although both cell types exhibited a similar pattern of diminished cell proliferation, CR-CSphCs showed a 3.9-fold increase in the half-maximal inhibitory concentration (IC50) (Figure 1A).

These results mirror the difficulties in targeting the CR-CSphCs subpopulation with therapeutically relevant doses of standard anti-cancer compounds that, in the meantime, comply with tolerability.

Given that high chemotherapy concentrations are required to induce significant inhibition of cell proliferation of CR-CSphCs, we sought to examine the anti-cancer effect of natural fractionated extracts of *Citrus sinensis* and *Humulus lupulus*, obtained from waste recovery by a time- and cost-saving method and containing Nobiletin and Xanthohumol as main components, respectively.

Thus, we performed class-specific isolation through reversed-phase semi-preparative liquid chromatography. As can be appreciated from Figure 1B–D, we collected polymethoxyflavones and prenylflavonoids from citrus and hop extracts, respectively. The identity of isolated metabolites was assessed by UHPLC-MS/MS analysis and supported by their retention times, comparing UV spectra and MS/MS data with those present in the literature.

In detail, the characteristic fragment ions of polymethoxyflavones were obtained by the loss of 31 Da corresponding to the CH3O group. Among them, compounds 3 and 5 were unambiguously identified as Nobiletin and Tangeretin, respectively, by comparison with the reference standards. Compound 1 was characterized as pentamethoxyflavone and identified as sinensetin while the chromatographic peaks 2 and 4 were identified as hexa- and heptamethoxyflavone, respectively. Compounds 6, 7, and 8, showed at *m*/*z* 367, *m*/*z* 369, and *m*/*z* 353 [M-H]- fragmentation ions at *m*/*z* 247, *m*/*z* 249, and at *m*/*z* 233 corresponding the product ions with the negative charge retained on the A-rings, following retro-Diels-Alder fragmentation [M-H-C8H8O]-.

Before assessing the effects of the Nobiletin-containing fraction (NCF), the Xanthohumol-containing fraction (XCF), and their combination (Mix) on tumor cells, we studied their impact on the viability of two healthy cell lines. The human stromal cells, HS-5, and the human umbilical vein endothelial cells, HUVEC, have been extensively described as reliable models to estimate and predict the side effects of anti-cancer drugs on healthy cells [44,45,46]. The two cell lines were treated with increasing concentrations of the two fractions for 24 and 48 h and only mild effects on cell viability were observed, supporting the absence of significant toxicity of Nobiletin and Xanthohumol (Figure S1A,B).

Together, these data indicate that NCF and XCF could serve as potential adjuvants of standard anti-cancer compounds, while minimizing the occurrence of side effects.

### 3.2. Phytoextracts Sensitize CR-CSphCs to Chemotherapy

In order to assess the potential use of either NCF, XCF, or their combination as chemo-sensitizing agents, we studied the potential synergistic effects of these natural compounds with FOX.

**Figure 2:**
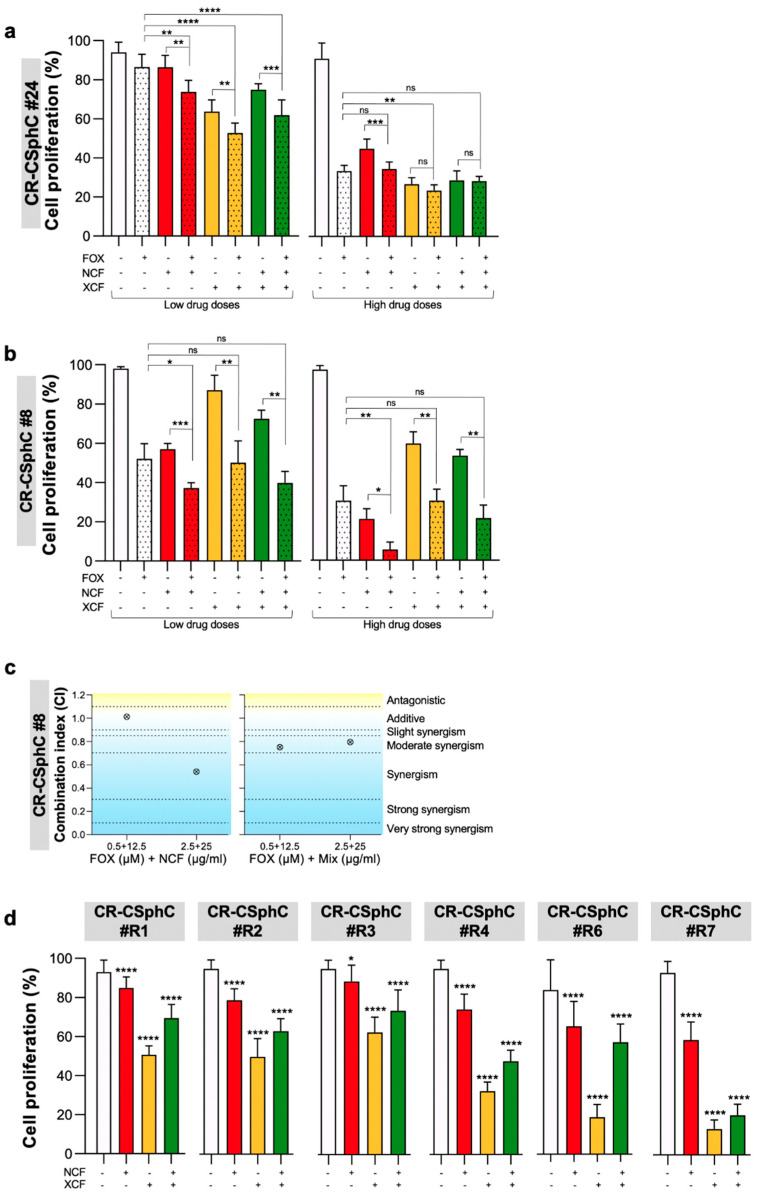
Combination of NCF plus chemotherapy hampers the proliferation of CR-CSphCs. (**a**) Cell proliferation percentage of CR-CSphCs (#24) treated with 25 or 40 µg/mL of NCF, XCF, or Mix extracts alone or in combination with 1.25 or 5 µM FOX for 48 h. Data are represented as mean ± SD of three independent experiments. Comparisons between two groups were made using a two-tailed Student’s *t*-test: ns, not significant, ** *p* ≤ 0.01, *** *p* ≤ 0.001, **** *p* ≤ 0.0001; (**b**) cell proliferation percentage of CR-CSphCs (#8) treated with 12.5 or 25 µg/mL of NCF, XCF, or Mix extracts alone or in combination with 0.5 or 2.5 µM FOX for 48 h. Data are represented as mean ± SD of three independent experiments. Comparisons between two groups were made using a two-tailed Student’s *t*-test: ns, not significant, * *p* ≤ 0.05, ** *p* ≤ 0.01, *** *p* ≤ 0.001; (**c**) synergy plot representing the combination index (CI), computed in CompuSyn by using the Chou–Talalay method, calculated from cell proliferation data of CR-CSphCs (#8) treated with different FOX and NCF and Mix dose pair at 48 h; (**d**) cell viability of metastatic CR-CSCs exposed for 72 h to 40 µg/mL NCF, XCF, or both extracts (Mix), as compared to control (vehicle). * *p* ≤ 0.05, **** *p* ≤ 0.0001.

Multiple lines of chemotherapy, after an initial tumor shrinkage, led to the selection and expansion of the CSC compartment with consequent tumor recurrence. We took advantage of our collection of CR-CSphCs isolated from both naïve primary CRC and liver metastasis of patients who were refractory to chemotherapy (Table S1). These cells represent a solid pre-clinical model, able to reproduce a patient’s sensitivity to drugs. Specifically, we selected three concentrations of extracts, 12.5µg/mL, 25µg/mL, and 40µg/mL, to test their effects on CR-CSphCs proliferation. The administration of NCF, XCF, and their combination (Mix) significantly reduced the proliferation of CR-CSphCs, including chemotherapy-resistant cells (Figure 2A–D and Figure S2). Combined exposure to NCF and FOX reduced the viability of six CR-CSphCs (#8, #24, #R1, #R2, #R3, and #R4), while treatment with FOX in combination with XCF significantly reduced the viability of four CR-CSphCs (#R7p, #24, #37, and #R2) (Figure S2A–C). Likewise, the combination index (CI) analysis, calculated by the Chou–Talalay method, highlighted the synergistic effects of Nobiletin and Mix plus FOX in reducing CR-CSphC#8 viability (Figure 2C and Figure S2A). These results were also validated on CRC cell lines, which showed a remarkable decrease in cell proliferation following the administration of the combined treatments (Figure S2E,F).

### 3.3. Nobiletin and Xanthohumol Induce Apoptosis of CR-CSphCs in Combination with Chemotherapy

**Figure 3:**
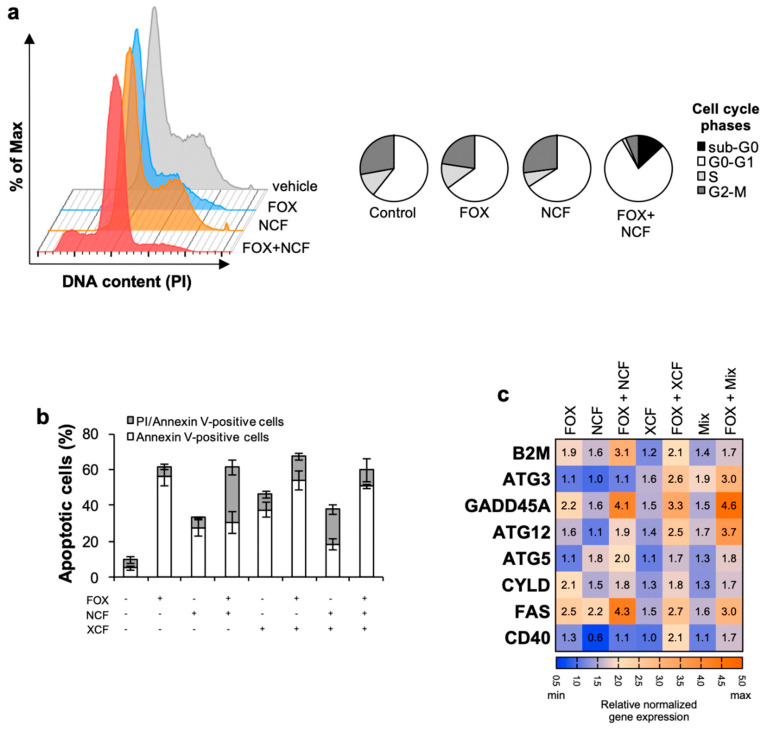
Flavonoids extracts plus chemotherapy induce apoptosis in CR-CSphCs. (**a**) Phase Representative flow cytometry analysis of cell cycle phases distribution in CR-CSphCs (#8) exposed to 0.5 µM FOX and 12.5 µg/mL NCF, alone or in combination, for 48 h. DNA content was assessed by propidium iodide (PI) staining; (**b**) percentage of apoptotic CR-CSphCs (#8) treated with 0.5 µM FOX and 12.5 µg/mL NCF, XCF, or both extracts, alone or in combination, for 48 h. The analysis was performed by flow cytometry on CR-CSphCs labeled with propidium iodide (PI) and Annexin-V; (**c**) gene expression analysis of pro-apoptotic genes in CR-CSphCs (#8) after exposure to 0.5 µM FOX and 12.5 µg/mL NCF, XCF, or both extracts (Mix), as compared to control (vehicle) for 48 h. Data are expressed as 2^−ΔΔCt^ expression values normalized to *GAPDH* and *HPRT* genes.

The genotoxic stress dictated by the extracts plus chemotherapy caused a reduction in CR-CSphCs S- and G2/M- cell cycle phases and substantially increased the G0/G1 and sub-G0 phase (Figure 3A and Figure S3A). The cytostatic effect caused by the combined treatment is conceivably induced by the short-term exposure of 48 h, which concomitantly allows early events of apoptosis. Thus, these data suggest that long-term treatments are required to definitively commit cells to apoptosis [29].

Accordingly, the combination of treatments induced the apoptotic events in CR-CSphCs, as demonstrated by the increased number of AnnexinV-positive cells (Figure 3B and Figure S3B).

To further confirm that NCF, XCF, and Mix are required to induce programmed cell death in combination with chemotherapy in CR-CSphCs, we analyzed the expression of genes related to apoptosis and autophagy. Gene expression analysis revealed enhanced expression of eight proapoptotic genes, ATG3, ATG5, ATG12, B2M, CD40, CYLD, FAS, and GADD45A, up-regulated in CR-CSphCs treated with each of the extracts and chemotherapy (Figure 3C). Flavonoids induce apoptosis at early events, which was paralleled with an increase in the expression of genes associated with apoptosis without determining a significant increase in Caspase-3 activity (Figure S3B), likely due to a caspase-3 independent apoptosis phenomenon.

These results suggest that fractionated extracts lessen the common dose of chemotherapeutic drugs, thereby reducing their side effects and rendering the therapeutic regimen more acceptable.

### 3.4. NCF and XCF Counteract Stemness Features of CR-CSCs

We and others demonstrated that standard chemotherapy efficaciously kills differentiated cells while sparing cells with stemness features [39]. Thus, we hypothesized that the addition of the flavonoid extracts to FOX chemotherapy could affect the stem compartment of CR-CSphCs.

**Figure 4:**
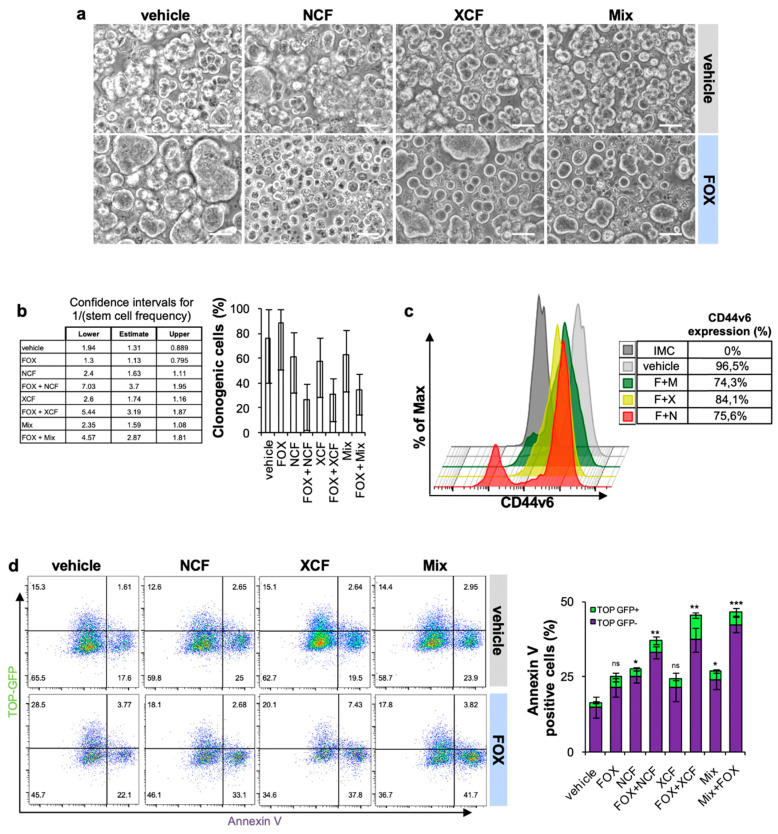
Flavonoids extracts plus chemotherapy decreased the stemness features of CR-CSphC. (**a**) Phase contrast image showing the sphere forming capability of CR-CSphCs treated with 0.5 µM FOX in combination with 12.5 µg/mL NCF, XCF, or both extracts (Mix), for 48 h; (**b**) clonogenic capability, evaluated by ELDA software, of CR-CSphCs. Pre-treated for 48 h as in (**a**); (**c**) representative flow cytometry analysis of CD44v6 expression on CR-CSCs following 48 h treatment as in (**a**); (**d**) representative flow cytometry analysis of Annexin V on CR-CSphCs (#8) transduced with TOP-GFP and treated as in A (left panel) and relative percentage of Annexin V positive CR-CSphCs (#8) (right panel). Data are expressed as mean ± SD of three independent experiments. Comparisons between two groups were made using a two-tailed Student’s *t*-test: ns, not significant, * *p* ≤ 0.05, ** *p* ≤ 0.01, *** *p* ≤ 0.001.

Therefore, we assessed the sphere-forming potential of CR-CSphCs as a functional approach to study CSCs self-renewal. Interestingly, following a short time exposure, we observed that FOX plus NCF, and to a lesser extent XCF and Mix, were able to suppress CR-CSCs sphere formation (Figure 4A and Figure S4A,B). In addition, we performed an in vitro limiting dilution assay to evaluate the residual self-renewal activity of cells previously exposed to 48 h of treatment. Consistent with previous results, the administration of extracts and chemotherapy hampered the clonogenic potential of CR-CSphC (Figure 4B).

We previously demonstrated that CD44v6 is a marker of CR-CSCs endowed with metastatic potential, and thereby responsible for disease progression [5]. After 48-h exposure to the combination of NCF, XCF, and Mix plus FOX, CR-CSCs showed a significantly lower expression of CD44v6 (75.6%, 84.1%, and 74.3%, respectively) compared to the control (96.5%) (Figure 4C and Figure S4C,D).

Wnt pathway activity is a functional biomarker that defines the CR-CSC population [7], and it is associated with poor prognosis in CRC patients [47]. Wnt/β-catenin activity is mostly associated with high expression levels of CD44v6, which significantly correlates with the overall survival probability of CRC patients [5]. Moreover, besides the well-known role of the Wnt pathway in the mechanisms that establish the stemness, Wnt gene targets were found involved at various levels in drug resistance [48]. To corroborate the role of flavonoid-based therapies in counteracting Wnt pathway activation, we analyzed the expression of Wnt pathway inhibitors and effectors. This therapeutic approach, which combines chemotherapy plus NCF and/or XCF was able to boost the gene expression levels of the major inhibitor of the Wnt pathway, DKK1, and lower the expression of the Wnt pathway effectors WNT3A, WNT5B, and WNT7B (Figure S4E,F) [49,50,51].

The activity of the Wnt pathway was validated by transducing CR-CSphCs with a Wnt reporter construct, which encodes for GFP when β-catenin binds to TCF/LEF promoter [7]. Wnt-high (GFP-positive) cells were sensitive to FOX in combination with extracts (Figure 4D). We also noticed an increase of Wnt-low early apoptotic cells positive for Annexin V. Thus, flavonoids treatment leads to a significant decrease of CD44v6 expression along with a reduced number of CR-CSCs bearing activation of the Wnt pathway.

## 4. Discussion

The pharmacological use of chemotherapy as a cancer remedy has been limited by its notorious side effects caused by toxicity to normal cells [17]. Thus, avoiding the undesired toxicity without compromising the targeting of cancer cells represents the main goal to be achieved in cancer treatment. Natural biofunctional molecules are known to exert their tumor-preventive effects by antioxidant polyphenols such as flavonoids [27,28]. Flavonoids, as natural compounds, play a crucial role in preventing intestinal barrier damage by preserving its integrity and the mucosal architecture [52], suggesting that they may not have any side effects on normal intestinal stem cells.

Both chemotherapy and targeted therapy, if not successful in eradicating the disease, may result in the reappearance of even more aggressive cancers. CR-CSCs expansion can occur during or after therapy interruption due to microenvironmental cues and/or the accumulation of genetic aberrations that nullify the effect of therapy [4].

Nobiletin and Xanthohumol, either singularly or 50% mixed with each other, synergize with FOX-based chemotherapy in order to reduce cell CR-CSphCs viability, the cell cycle S phase and G2/M phase, the sphere-forming capability, and clonogenic potential. Notably, flavonoids treatment lowers the number of CR-CSCs expressing high levels of Wnt and CD44v6, which together with the Wnt pathway activity is the most accurate functional biomarker that identifies a CR-CSC population endowed with metastatic potential [5].

Nobiletin has been reported to sensitize cells to chemotherapy and promote apoptosis in CRC cell lines [53]. Likewise, Xanthohumol has also been reported to sensitize CRC cells to chemotherapy by inhibiting cell proliferation and induce DNA damage and apoptosis in CRC cells [30]. In accordance, NCF and XCF in combination with FOX determine upregulation of pro-apoptotic genes, as well as downregulation of anti-apoptotic genes. Furthermore, given that several of the pro-apoptotic genes induced by flavonoids and the FOX combined treatment, such as ATG3, ATG5, and ATG12, play a critical role in the autophagic process [54,55], we hypothesize that CR-CSphCs exposed to the combined treatment experience severe intra-cellular stress, leading to apoptosis that may follow an autophagic process. Interestingly, we observed that both NCF and XCF, albeit showing cytotoxic effects toward cancer cells, are well tolerated by normal cells. This phenomenon is still not thoroughly understood and could be explained by the presence of altered metabolism or activation of molecular pathways exclusively in cancer cells.

The underlying mechanisms of Nobiletin and Xanthohumol in reducing the viability of CR-CSphCs refractory to chemotherapy is possibly related to the downregulation of stemness programs, such as the Wnt pathway. Indeed, it has already been demonstrated that chemotherapy, for example, 5-FU, induces the activation of the Wnt pathway in CRC cells to mediate treatment escape [56].

Notably, beyond Wnt pathway activation, other stemness and survival pathways specific for CSCs have been described to be involved in the anticancer activity of Nobiletin and Xanthohumol [57,58]. Flavonoids decrease the expression of drug efflux transporters in CRC, which is a well-described mechanism of drug resistance in CSCs [59,60]. This could represent a potential mechanism for the specific targeting of the CR-CSC subset.

In summary, we investigated how NCF and XCF interfere with cell proliferation and apoptosis, by possibly targeting stemness pathways involved in the onset and progression of cancer. Interestingly, we found that these plant derived-natural compounds, either singularly or in combination, are beneficial as additive molecules to chemotherapy, possibly limiting anti-cancer cytotoxicity towards normal cells. The novelty of our research relies on the synergistic/additive effect of the Nobiletin/Xanthohumol treatment in combination with chemotherapy, which affects the viability of CR-CSCs purified and propagated from CRC liver metastasis of patients treated with chemotherapy. In conclusion, flavonoids could serve as a promising strategy for anti-cancer treatment, which preserves patients’ quality of life. 

## 5. Conclusions

Here we investigated the potential effects of natural flavonoids, both polymethoxyflavones and prenylflavonoids, as potential adjuvants in CRC therapy. The results obtained showed how specific fractions from natural extracts are able to improve the efficacy of chemotherapy, at the same time reducing cancer cell survival and chemotherapeutics cytotoxicity. Specifically, these fractions are characterized by the presence of active compounds that have been previously characterized for their anticancer potential, namely Nobiletin form *Citrus sinensis* and Xanthohumol from *Humulus lupulus*. The obtained results will pave the way for further investigations about the use of fractionated natural extracts as adjuvants in chemotherapy in the form of functional or fortified foods and/or dietary supplements.

## Data Availability

Data related to the study are included in the article or uploaded as supplementary information. Data are available from the corresponding authors (P.C. and G.S.) upon reasonable request.

## Supplementary Materials

The following are available online at https://www.mdpi.com/article/10.3390/cancers13163927/s1, Figure S1: NCF and XCF do not affect non-transformed cells. Figure S2: NCF and XCF sensitize cancer cells to chemotherapy. Figure S3: NCF and XCF plus chemotherapy induce apoptosis in CR-CSphCs. Figure S4: NCF and XCF plus chemotherapy counteract the stemness potential of CR-CSCs. Table S1: CR-CSphCs characterization, CD44v6 expression, MSI profile, and *KRAS, BRAF, APC,* and *PIK3CA* gene mutation profile.
