# Supplementary material for: Nobiletin and Xanthohumol Sensitize Colorectal Cancer Stem Cells to Standard Chemotherapy
Source: Cancers (Basel). 2021 Aug 4;13(16):3927. doi: 10.3390/cancers13163927 (PMC8392547; doi:10.3390/cancers13163927)
Supplement: Supplementary file 1 [file cancers-13-03927-s001.zip › cancers-1281252-supplementary/cancers-1281252-SI.pdf]

Nobiletin and Xanthohumol Sensitize Colorectal Cancer Stem Cells to Standard Chemotherapy

Alice Turdo, Antonino Glaviano, Giacomo Pepe, Federica Calapà, Stefania Raimondo, Micol Eleonora Fiori, Daniela Carbone, Manuela Giovanna Basilicata, Veronica Di Sarno, Carmine Ostacolo, Barbara Parrino, Stella Cascioferro, Camilla Pecoraro, Simone Di Franco, Diana Bellavia, Miriam Gaggianesi, Veronica Veschi, Melania Lo Iacono, Gloria Ganduscio, Vincenzo Davide Pantina, Laura Rosa Mangiapane, Maria Rita Bongiorno, Riccardo Alessandro, Matilde Todaro, Ruggero De Maria, Patrizia Diana, Pietro Campiglia and Giorgio Stassi

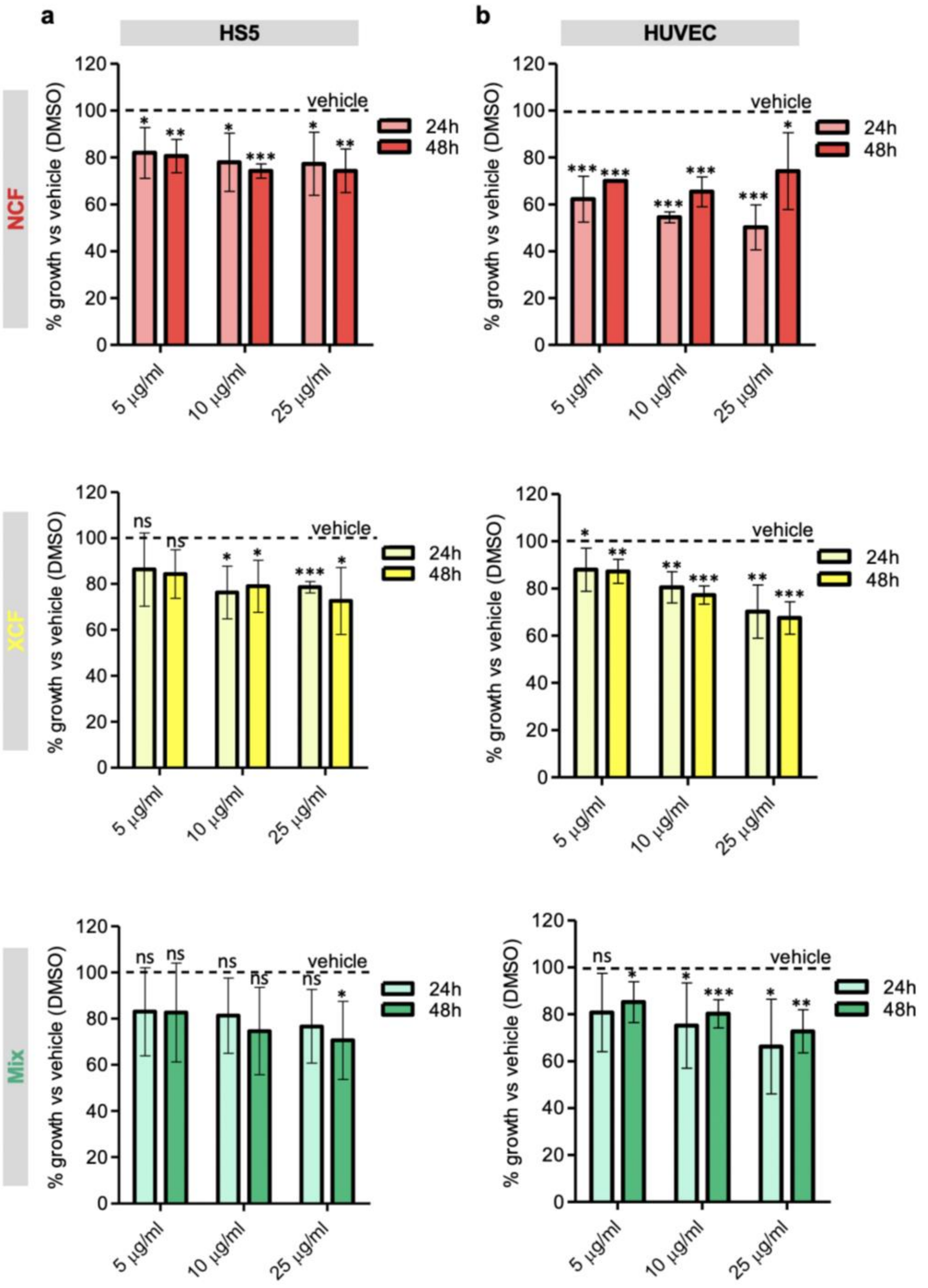

Figure S1. NCF and XCF do not affect non-transformed cells. Percentage of growth of HS5 (**a**) and HUVEC (**b**) cell lines treated with 5, 10, and 25 µg/ml of NCF, XCF, or the Mix of extracts for 24 and 48 hours. Values are plotted as the percentage of 5 growth versus the vehicle (DMSO, dotted line). Data are represented as means ± SD. Comparisons between two groups (cells treated with the extracts vs cells treated with the vehicle) were made using a two-tailed Student's t-test: *$p \leq 0.05$, **$p \leq 0.01$, ***$p \leq 0.001$.

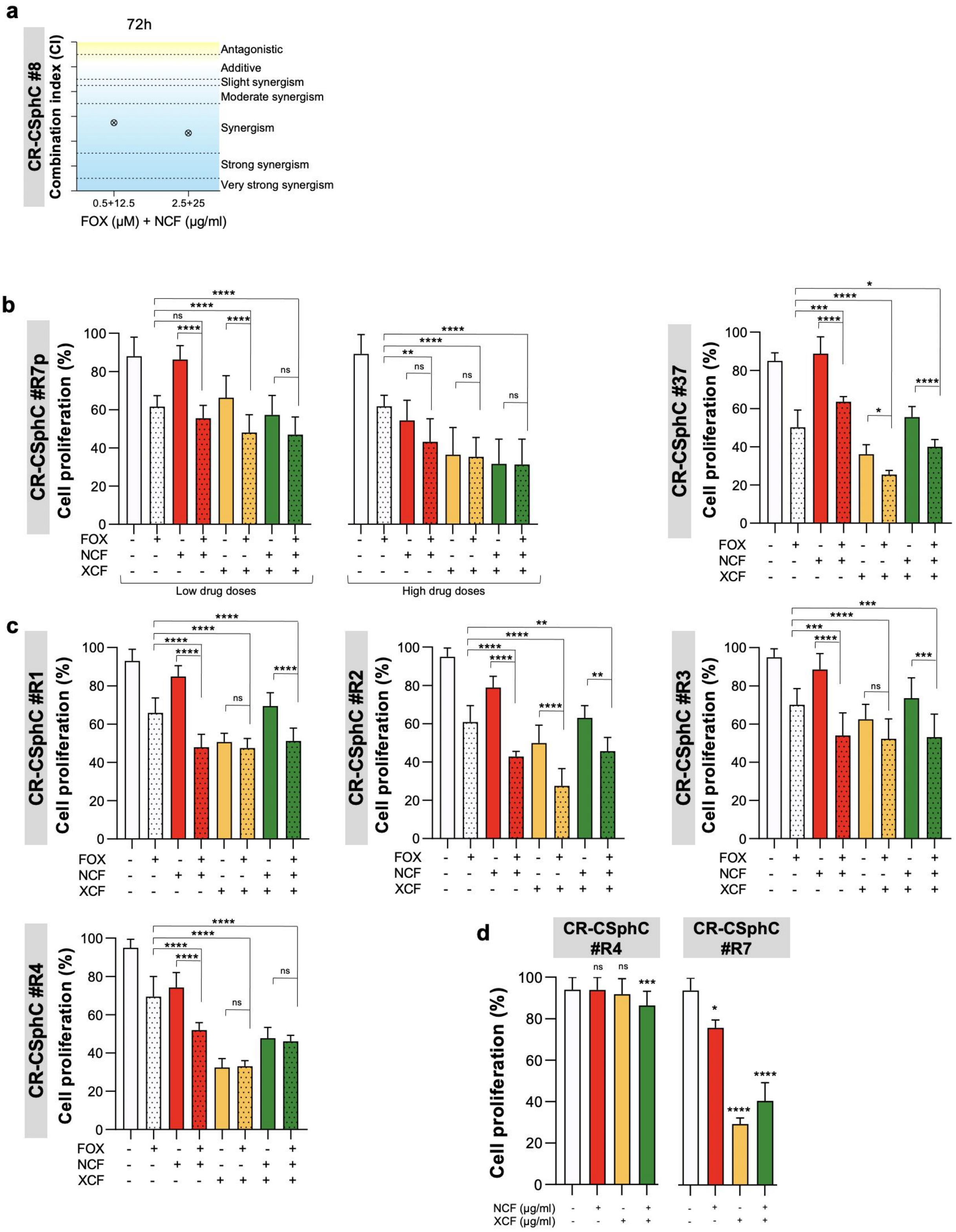

a
72h
CR-CSphC #8
Combination index (CI)
Antagonistic
Additive
Slight synergism
Moderate synergism
Synergism
Strong synergism
Very strong synergism
0.5+12.5
2.5+25
FOX (μM) + NCF (μg/ml)
b
CR-CSphC #R7p
Cell proliferation (%)
FOX
NCF
XCF
Low drug doses
High drug doses
CR-CSphC #37
c
CR-CSphC #R1
CR-CSphC #R2
CR-CSphC #R3
CR-CSphC #R4
d
CR-CSphC #R4
CR-CSphC #R7
NCF (μg/ml)
XCF (μg/ml)

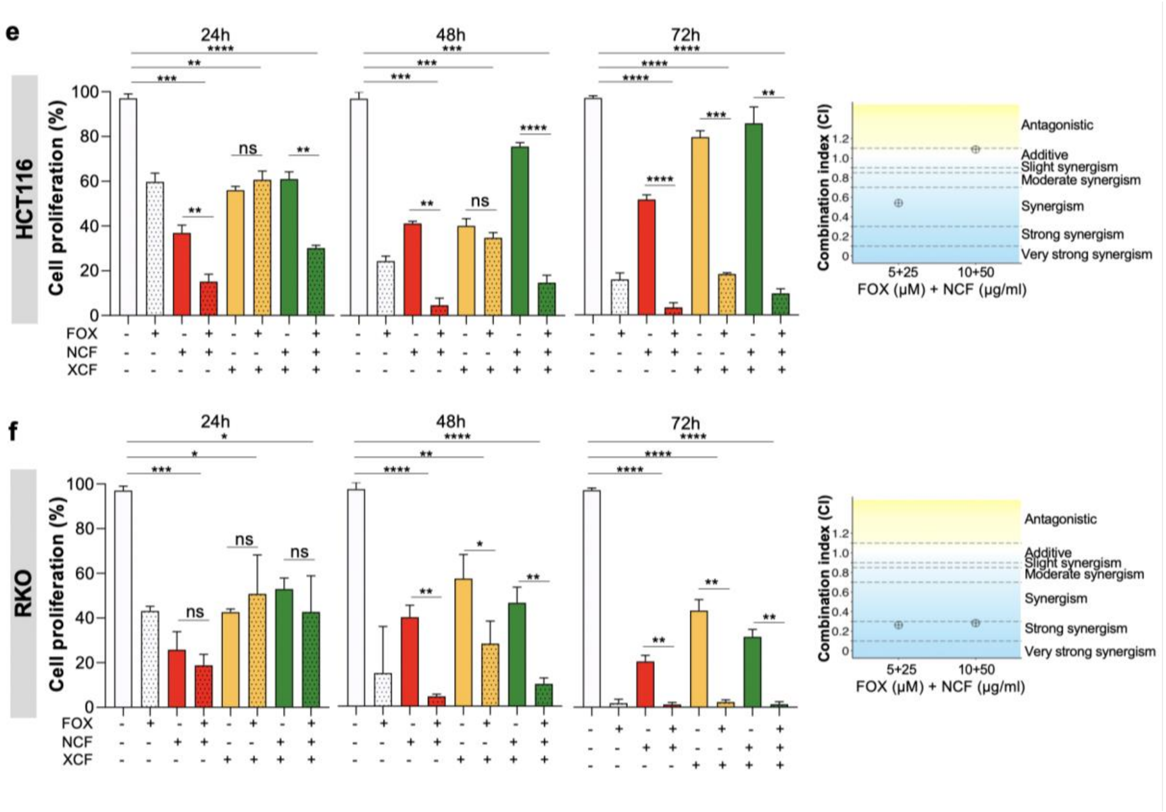

Figure S2. NCF and XCF sensitize cancer cells to chemotherapy (**a**) Synergy plot representing the combination index (CI), computed in CompuSyn by using Chou-Talalay method, calculated from cell proliferation data of CR-CSphCs (#8) treated with different FOX and NCF dose pair at 72 hours; (**b**) Cell proliferation percentage of primary CR-CSphCs (#R7p) treated with 25 and 40 µg/ml of NCF, XCF or Mix extracts alone or in combination with 5 µM FOX for 72 hours (left panel) and CR-CSphCs (#37) treated with 25 µg/ml of NCF, XCF or Mix extracts alone or in combination with 1.25 µM FOX for 72 hours (right panel); (**c**) Cell proliferation percentage of CR-CSC #R1, #R2, #R3, #R4 treated with 40 µg/ml of NCF, XCF or Mix extracts alone or in combination with 5 µM FOX for 72 hours; (**d**) Cell proliferation percentage of CR-CSC #R4 and #R7 treated with 25 µg/ml of NCF, XCF or Mix extracts for 72 hours; (**e,f**) Cell viability of CRC cell lines (HCT116 and RKO) treated with 25 µg/ml of NCF, XCF or Mix extracts alone or in combination with 5 µM FOX at the indicated time points (left panel). Synergy plot representing the combination index (CI), computed in CompuSyn by using Chou-Talalay method, calculated from cell proliferation data of CRC cell lines treated with different FOX and NCF dose pair at 48 hours (right panel). Data are represented as mean ± SD of three independent experiments. Percentage of untreated control (vehicle) is shown. Comparisons between two groups were made using a two-tailed Student's t-test: ns, not significant, *$p \leq 0.05$, **$p \leq 0.01$, ***$p \leq 0.001$, ****$p \leq 0.0001$.

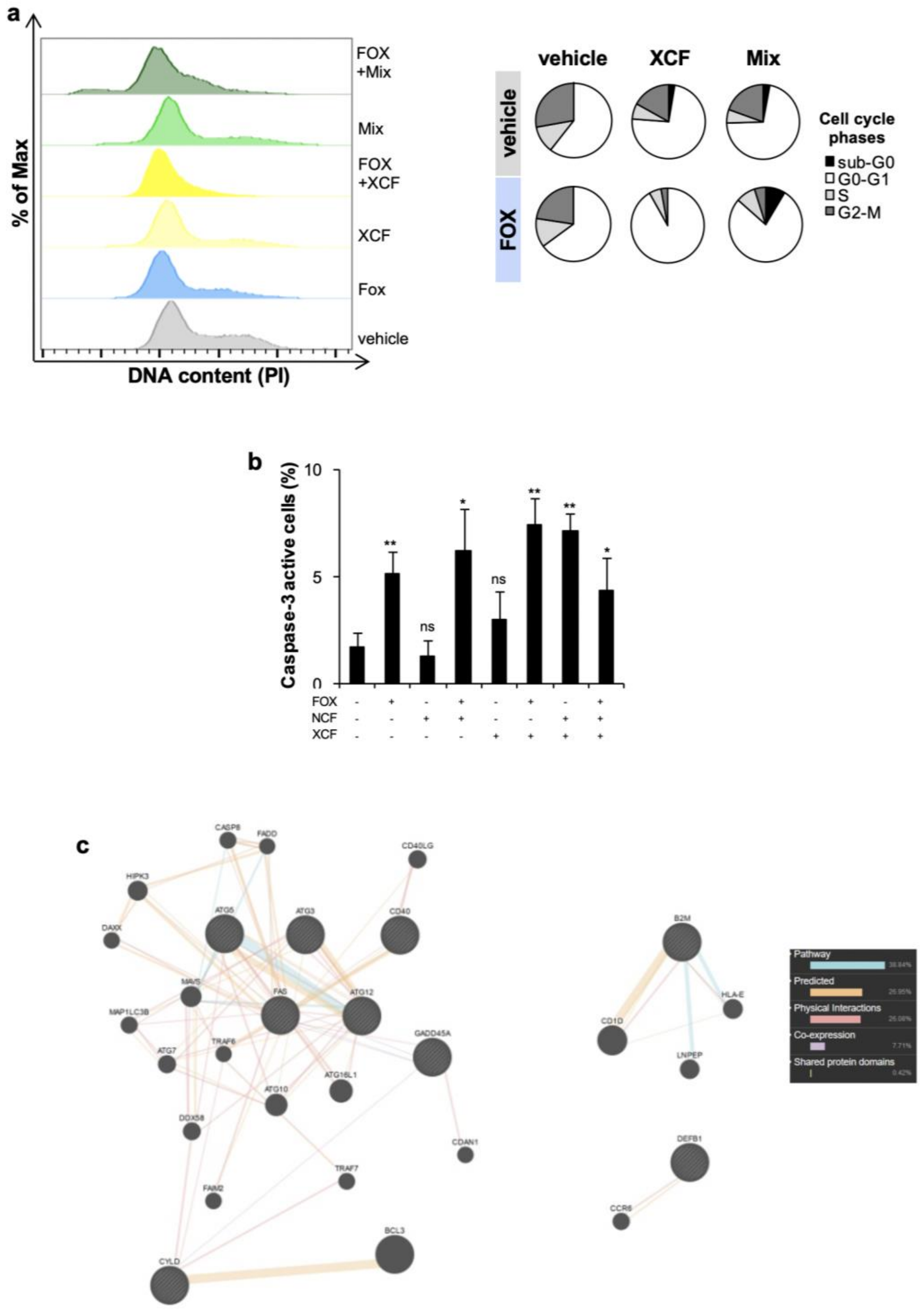

Figure S3. NCF and XCF plus chemotherapy induce apoptosis in CR-CSphCs (**a**) Representative flow cytometry analysis of cell cycle phases distribution in CR-CSphCs (#8) exposed to 0.5 μM FOX and 12.5 μg/ml Xanthohumol or Mix, alone or in combination, for 48 hours. DNA content was assessed by propidium iodide (PI) staining; (**b**) Percentage of cells treated as in (a) showing caspase-3 activity assessed by flow cytometry analysis; (**c**) Network integration of multiple genes showed in Figure 3C calculated by geneMANIA software.

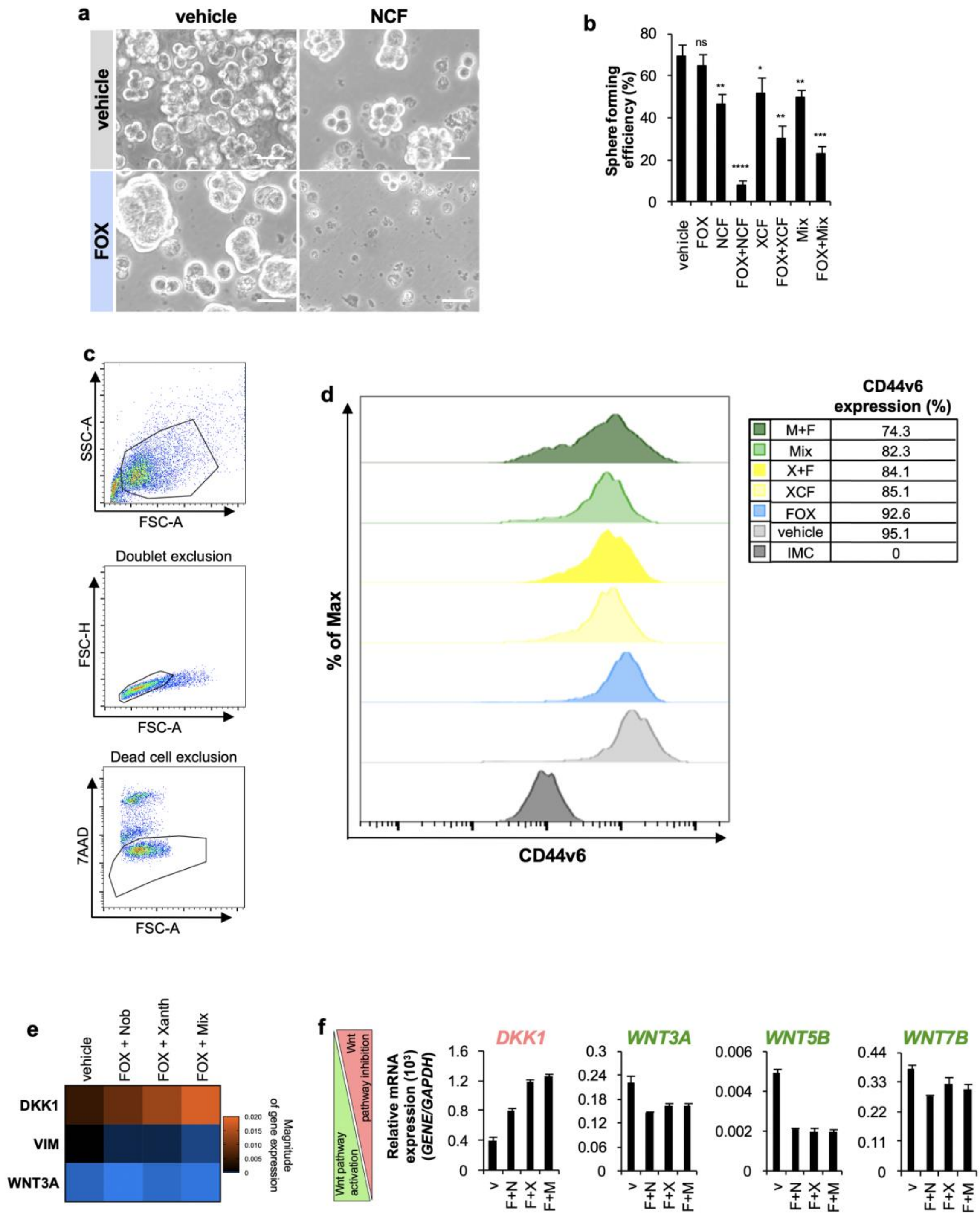

Figure S4. NCF and XCF plus chemotherapy counteract the stemness potential of CR-CSCs (**a**) Phase contrast image showing the sphere forming capability of CR-CSCs, cultured at low cell density, treated with 0.5 µM FOX and 12.5 µg/ml NCF, alone or in combination, for 48 hours, scale bar represents 25µm; (**b**) Sphere forming efficiency of CR-CSCs treated with 0.5 µM FOX and 12.5 µg/ml NCF, alone or in combination, for 48 hours (**c**) Representative flow cytometry analysis showing the gating strategies of CR-CSphC shown in Figure 3D; (**d**) Representative flow cytometry analysis of CD44v6 expression on CR-CSCs following 48 hours treatment with 12.5 µg/ml of NCF, XCF or Mix extracts alone or in combination with 0.5 µM FOX for 72 hours; (**e**) Gene expression analysis of genes involved in stemness and EMT in CR-CSphCs after exposure to 0.5 µM FOX and 12.5 µg/ml NCF, XCF or Mix, as compared to control (vehicle) for 48 hours. Data are expressed as $2^{-\Delta\Delta Ct}$ expression values normalized to *GAPDH* and *HPRT* genes; (**f**) Relative mRNA expression levels of genes involved in Wnt pathway activation (*DKK1*, *AXIN1*, *AXIN2*, *CK1*) and inhibition (*WNT3A*, *WNT5B*, *WNT7B*). *GAPDH* was used as control.

	#	Age	Gender	Site	Stage	Grading	TNM classification	MSI clinical relevant	CD44v6 (%)	*KRAS*	*BRAF*	*APC*	*PIK3CA*
CR-CSphC	3	85	F	colon (right site)	IIIC	G3	T3N2M0	MSI	33	*wt*	*mut*	*wt*	*mut*
	8	57	F	colon (right site)	IV	G3	T3N2M1	MSS	92.3	*mut*	*wt*	*mut*	*mut*
	24	51	F	colon (right site)	IIA	G2	T3N0M0	MSI	9.96	*wt*	*wt*	*mut*	*wt*
	37	82	M	colon (right site)	IIIC	G3	T3N2M0	MSI	46	*wt*	*wt*	*mut*	*wt*
	59	78	M	colon	IIIB	G3	T3N1M0	MSS	85.4	*mut*	*wt*	*wt*	*wt*
	R7p	70	M	colon (left side)	NA	NA	NA	NA	NA	*wt*	*mut*	NA	*wt*
Chemoresistant CR-CSphC	R1	65	M	liver metastasis of colon cancer (sigmoid)	IIA	NA	T3N0M0	MSS	13.7	*wt*	*wt*	NA	NA
	R2	61	F	liver metastasis of colon cancer (rectum)	IV	NA	T3N1M1	MSS	19.1	*wt*	*wt*	NA	NA
	R3	62	F	liver metastasis of colon cancer (rectum)	IIIB	NA	T3N1M0	MSS	22.8	*mut*	*wt*	NA	NA
	R4	38	M	liver metastasis of colon cancer (rectum)	IV	NA	T4N1M1	MSS	93	*wt*	*mut*	NA	NA
	R6	69	F	liver metastasis of colon cancer (left site)	NA	NA	NA	NA	NA	*wt*	*wt*	NA	*wt*
	R7	70	M	liver metastasis of colon cancer (left site)	NA	NA	NA	NA	NA	*wt*	*mut*	NA	*wt*

Table S1. CR-CSphCs characterization, CD44v6 expression, MSI profile, and *KRAS*, *BRAF*, *APC* and *PIK3CA* gene mutational profile. TNM classification is referred to the time of initial diagnosis. Wt, wild-type; mut, mutated; NA, data not available.
